# Supplementary material for: Giant electric field-induced second harmonic generation in polar skyrmions
Source: Nat Commun. 2024 Feb 14;15:1374. doi: 10.1038/s41467-024-45755-5 (PMC10866987; doi:10.1038/s41467-024-45755-5)
Supplement: Supplementary file 1 — Supplementary Information [file 41467_2024_45755_MOESM1_ESM.pdf]

Supplementary Information for Giant electric field-induced second harmonic generation in polar skyrmions

Sixu Wang[1,#], Wei Li[1,#], Chenguang Deng[1], Zijian Hong[2,3,*], Han-Bin Gao[4], Xiaolong Li[5], Yueliang Gu[5], Qiang Zheng[4,*], Yongjun Wu[2], Paul G. Evans[6], Jing-Feng Li[1], Ce-Wen Nan[1], Qian Li[1,*]

1. State Key Laboratory of New Ceramics and Fine Processing, School of Materials Science and Engineering, Tsinghua University, Beijing, 100084, China
2. School of Materials Science and Engineering, Zhejiang University, Hangzhou, 310027, China
3. Research Institute of Zhejiang University-Taizhou, Taizhou, Zhejiang 318000, China
4. CAS Key Laboratory of Standardization and Measurement for Nanotechnology, CAS Center for Excellence in Nanoscience, National Center for Nanoscience and Technology, Beijing, 100190, China.
5. Shanghai Synchrotron Radiation Facility, Shanghai Advanced Research Institute, Chinese Academy of Sciences, Shanghai, 201204, China
6. Department of Materials Science and Engineering, University of Wisconsin-Madison, Madison, 53706, United States

# These authors contribute equally to this work.

* Corresponding authors: qianli_mse@tsinghua.edu.cn (QL), zhengq@nanoctr.cn (QZ), hongzijian100@zju.edu.cn (ZH)

This file includes:

Figures S1 to S6

Supplementary Notes 1-5

References

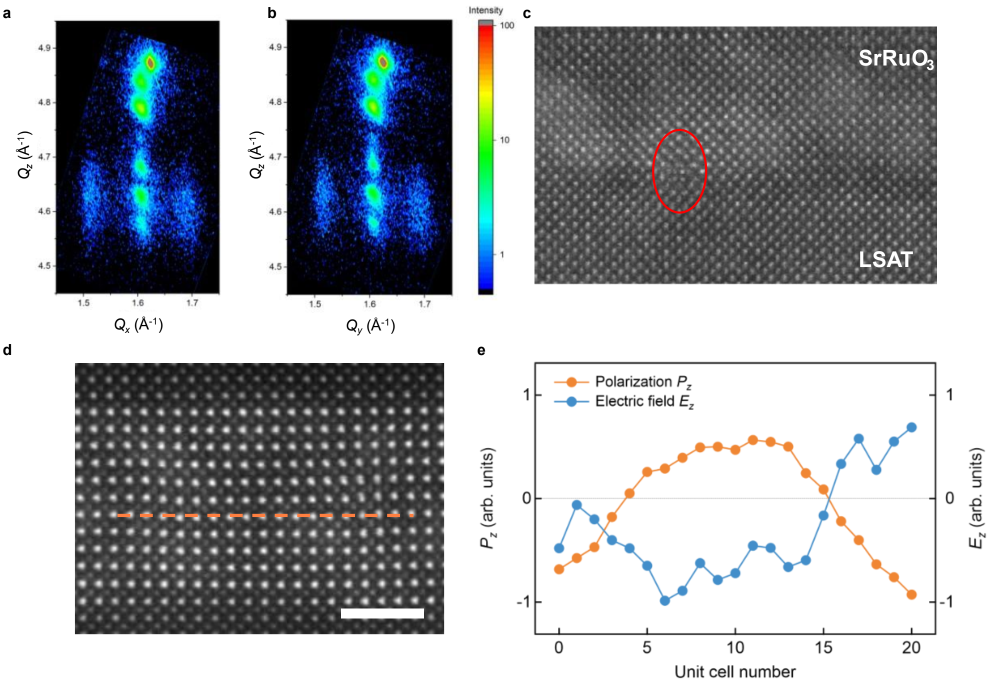

Figure S1 | Structural characterization of the superlattice film (SL). a,b, Laboratory XRD (Bruker D8 Discover, Cu $K_{\alpha 1}$ source) based reciprocal space maps of $[(PbTiO_3)_{14}/(SrTiO_3)_{16}]_8$ superlattice films around the LSAT 103 (a) and 013 (b) reflections. Different Q_x (Q_y) locations of the LSAT substrate and superlattice diffraction peaks indicate strain relaxation in the epitaxial superlattice films. **c**, Cross-sectional HAADF-STEM image about the interface between the bottom SRO electrode and the LSAT substrate. **d,** High-resolution HAADF image shown in Fig. 1e. The dashed line marks the center line of the $PbTiO_3$ layer. Scale bar = 2 nm. **e**, Line profiles of the z component of local polarization P_z and electric field E_z along the center line of $PbTiO_3$ layer marked in (d).

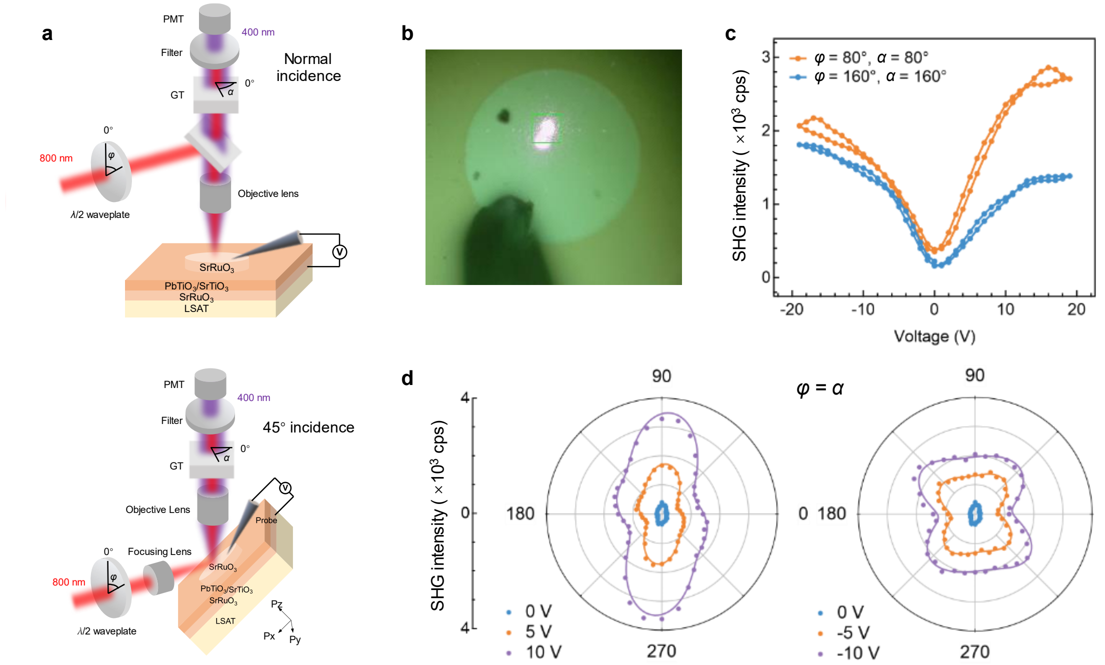

Figure S2 | *In situ* electric field induced SHG measurement under normal incidence. a, Schematic of the experimental setup. **b**, Optical picture of a $SrRuO_3$ top electrode attached with a tungsten probe tip (shadow). The laser spot can be observed to be focused on the electrode. **c**, SHG intensity as a function of applied voltage under different conditions of φ and α angles. **d**, Polarimetry plots of the SHG intensity measured in the α-φ angle coupled ($\alpha = \varphi$) scan mode for different applied voltages. The patterns are drawn under same intensity scales.

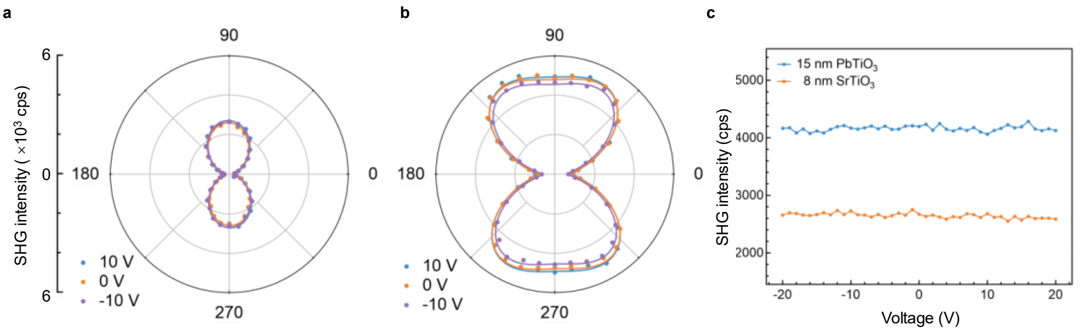

Figure S3 | *In situ* electric field induced SHG measurement on single layer films. a,b, Polarimetry plots of SHG intensity of single layer $SrTiO_3$ (a) and $PbTiO_3$ (b) films measured under the output polarization angle α = 90° (*s*-out) for different applied voltages. **c**, SHG intensity of single layer $PbTiO_3$ and $SrTiO_3$ as a function of external voltage. Both films were grown on $SrRuO_3$ buffered LSAT substrates. Both data curves exhibit no signs of increased SHG intensity under external electric field, thus no EFISH process occurring in such single-layer films of $PbTiO_3$ and $SrTiO_3$.

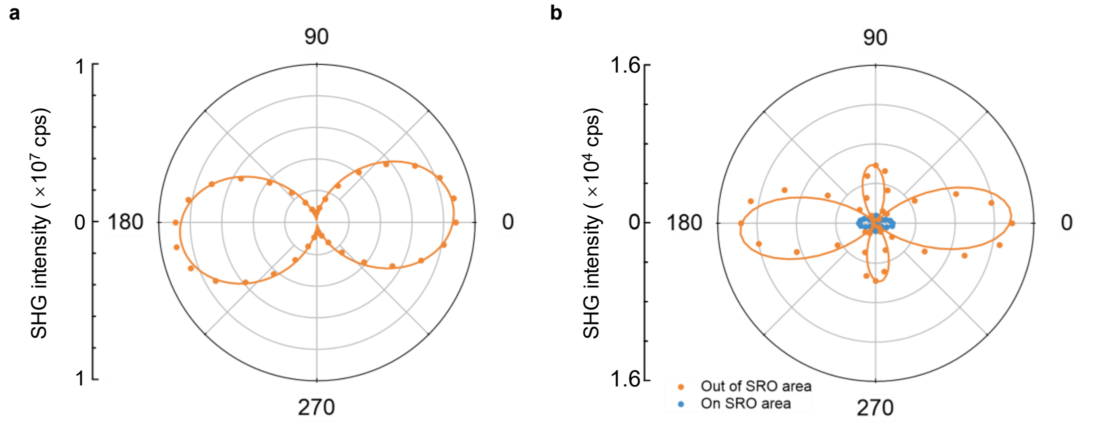

Figure S4 | Comparative SHG measurements for calculating the nonlinear susceptibility coefficient $\chi^{(2)}$ and SHG efficiency. a, SHG intensity measured on a 300 nm thick *x*-cut $LiNbO_3$ on insulator (LNOI) under the output polarization angle $\alpha = 0°$ (*p*-out). The average output power of the laser was 50 mW in this measurement. **b,** SHG intensities measured inside and outside the $SrRuO_3$ top electrode area on a single layer $PbTiO_3$ film under the output polarization angle $\alpha = 0°$ (*p*-out). The SHG intensity level inside the $SrRuO_3$ electrode is ~1/8 of the intensity level outside.

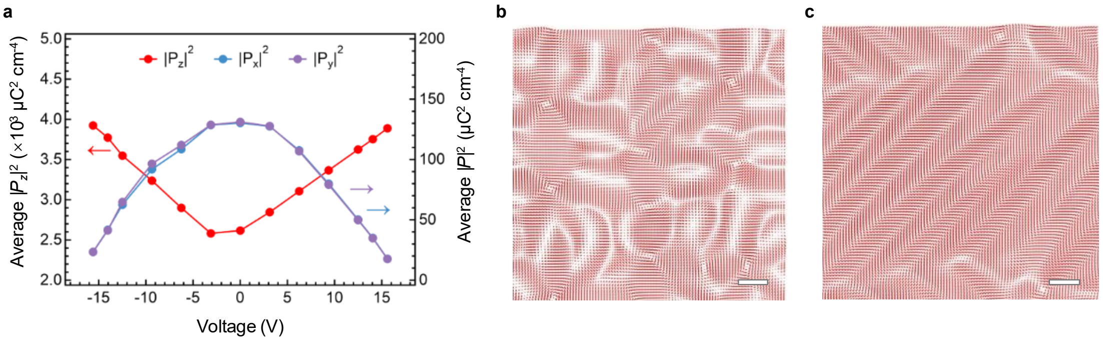

Figure S5 | Phase-field simulation results: **a,** Simulation box-averaged values of the three polarization components in the superlattice as a function of bias voltage. **b,c,** In-plane polarization vector map in a $PbTiO_3$ layer at 400 K (b) and 500 K (c) showing the disappearance of polar skyrmions. Scale bar = 8 nm.

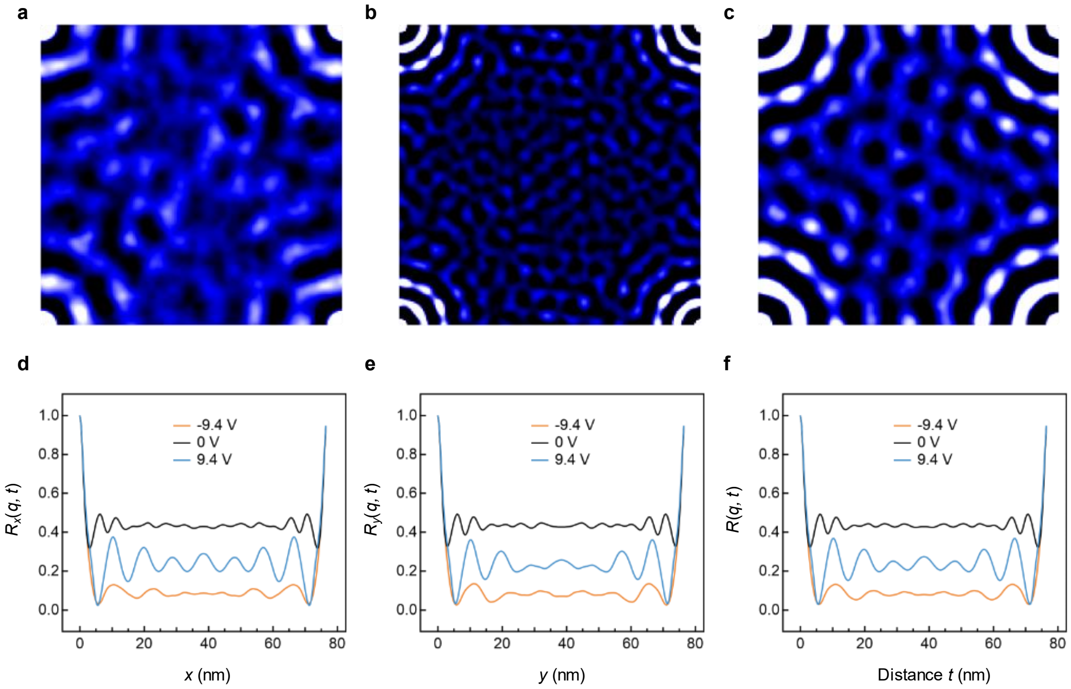

Figure S6 | a-c, Autocorrelation maps of Pontryagin density q for -9.4 V (a), 0 V (b) and 9.4 V (c) extracted from the phase-field models. **d-f,** The autocorrelation functions of the Pontryagin density $R(q, t)$, calculated based on the phase-field model results, as a function of pseudo-period distance along the x axis (d) and y axis (e), and the root mean square of both directions (f).

Supplementary Note 1 | Fitting of the SHG tensors for different polarities of external voltage.

SHG is dependent on the lattice structure (namely, the point group symmetry) of materials. We can express such nonlinear optical properties using a second-order nonlinear coefficient tensor as:

$$\begin{pmatrix} P_x(\varphi) \\ P_y(\varphi) \\ P_z(\varphi) \end{pmatrix} = \epsilon_0 \begin{pmatrix} \chi_{11} & \chi_{12} & \chi_{13} & \chi_{14} & \chi_{15} & \chi_{16} \\ \chi_{21} & \chi_{22} & \chi_{23} & \chi_{24} & \chi_{25} & \chi_{26} \\ \chi_{31} & \chi_{32} & \chi_{33} & \chi_{34} & \chi_{35} & \chi_{36} \end{pmatrix} \begin{pmatrix} E_x^2(\varphi) \\ E_y^2(\varphi) \\ E_z^2(\varphi) \\ 2E_yE_z(\varphi) \\ 2E_zE_x(\varphi) \\ 2E_xE_y(\varphi) \end{pmatrix} \quad \text{(S1)}$$

where $E_i(\varphi)$ is the electric field of the fundamental wave. For specific symmetries, certain elements in this tensor are zero, so that the tensor can be simplified with fewer non-zero elements. For example, for point group symmetries 4*mm* and *mm*2, only χ_{31}, χ_{32}, χ_{33}, χ_{15}, and χ_{24} are non-zero, and for 4*mm*, $\chi_{31} = \chi_{32}$ and $\chi_{15} = \chi_{24}$. The SHG intensity measured under *p*-out and *s*-out conditions as a function of the fundamental wave polarization angle φ can be given by $I_p(\varphi) = |P_x(\varphi)|^2$ and $I_s(\varphi) = |P_y(\varphi)|^2$, noting that only the P_x and P_y contribute to the intensity of light polarized in the *xy* plane.

In the case of non-zero angles of incidence, the coordinate systems of incident light, the sample and output light are different. Therefore, transformations between such coordinates are necessary. Defining E_i and $E_{i,s}$ as the electric field of the fundamental wave in the coordinate systems of incident light and the sample, $P_{i,s}$ and P_i as the nonlinear polarization in the coordinates of the sample and the output light, for point group symmetries 4*mm*, $P(\varphi)$ can be expressed as follows:

$$\begin{pmatrix} E_{x,s}(\varphi) \\ E_{y,s}(\varphi) \\ E_{z,s}(\varphi) \end{pmatrix} = \begin{pmatrix} \cos\gamma & 0 & \sin\gamma \\ 0 & 1 & 0 \\ -\sin\gamma & 0 & \cos\gamma \end{pmatrix} \begin{pmatrix} E_x(\varphi) \\ E_y(\varphi) \\ E_z(\varphi) \end{pmatrix} \quad \text{(S2)}$$

$$\begin{pmatrix} P_{x,s}(\varphi) \\ P_{y,s}(\varphi) \\ P_{z,s}(\varphi) \end{pmatrix} = \epsilon_0 \begin{pmatrix} 0 & 0 & 0 & 0 & \chi_{15} & 0 \\ 0 & 0 & 0 & \chi_{24} & 0 & 0 \\ \chi_{31} & \chi_{32} & \chi_{33} & 0 & 0 & 0 \end{pmatrix} \begin{pmatrix} E_{x,s}^2(\varphi) \\ E_{y,s}^2(\varphi) \\ E_{z,s}^2(\varphi) \\ 2E_{y,s}E_{z,s}(\varphi) \\ 2E_{z,s}E_{x,s}(\varphi) \\ 2E_{x,s}E_{y,s}(\varphi) \end{pmatrix} \quad \text{(S3)}$$

$$\begin{pmatrix} P_x(\varphi) \\ P_y(\varphi) \\ P_z(\varphi) \end{pmatrix} = \begin{pmatrix} \cos\gamma & 0 & \sin\gamma \\ 0 & 1 & 0 \\ -\sin\gamma & 0 & \cos\gamma \end{pmatrix} \begin{pmatrix} P_{x,s}(\varphi) \\ P_{y,s}(\varphi) \\ P_{z,s}(\varphi) \end{pmatrix} \quad \text{(S4)}$$

where γ is the incident angle, 45° in our case. Given the low numerical aperture of the focusing lens in the 45° incident-angle measurement, the focusing effect of incident light is ignored. It follows that $E_x(\varphi) = |E| \cos\varphi$, $E_y(\varphi) = |E| \sin\varphi$, $E_z(\varphi) = 0$, where E refers to the total electric field of the input fundamental wave. Therefore, for the 45° incidence, the measured SHG intensity can be expressed as:

$$I_p(\varphi) \propto \left(\frac{\sqrt{2}}{4}\chi_{31}\cos^2\varphi + \frac{\sqrt{2}}{2}\chi_{32}\sin^2\varphi + \frac{\sqrt{2}}{4}\chi_{33}\cos^2\varphi - \frac{\sqrt{2}}{4}\chi_{15}\cdot 2\cos^2\varphi\right)^2$$

(S5)

$$I_s(\varphi) \propto \left(\frac{\sqrt{2}}{2}\chi_{24}\cdot 2\cos\varphi\sin\varphi\right)^2 \quad \text{(S6)}$$

Eqs. S5 and S6 correspond to the polarization-angle dependent SHG intensity for the p-out and s-out conditions, respectively. Here, using the Global Fit function package of Igor Pro software, the relative ratios between the second-order nonlinear coefficients can be extracted as $|\chi_{31}| : |\chi_{32}| : |\chi_{33}| : |\chi_{24}| : |\chi_{15}| = 0.04 : 0.04 : 2.05 : 0.57 : 0.57$ for positive bias voltages, and $1 : 1 : 0.24 : 0.62 : 0.62$ for negative voltages. For negative voltage, the result that $\chi_{31} = \chi_{32}$ and $\chi_{15} = \chi_{24}$ conforms with the symmetry of point group 4*mm*, and the ratios between the χ_{31}, χ_{33}, and χ_{15} are close to those of $PbTiO_3$ bulk single crystal, indicating the transformation from the skyrmions to *c* domains. The observed trend of SHG intensity increasing with applied voltage can be explained by increasing part of the superlattices transforming to *c* domains. However, for positive applied voltage, a significantly higher χ_{33} has been observed, which is ~9 times the χ_{33} for negative voltages. This reveals a different route of transformation (described in the main text), and the extraordinarily large χ_{33} contributes to larger SHG intensities and higher modulation depths. The extracted SHG tensor coefficients were also cross-checked using the #SHAARP simulation package[1], whose output is in good agreement with the experimentally measured SHG intensities.

Supplementary Note 2 | Calculation of the optical nonlinear susceptibility coefficient and SHG efficiency.

The SHG intensity can be expressed as

$$I_{2\omega} \propto \frac{l^2\chi^2}{n_\omega^2 n_{2\omega}} I_\omega^2 \quad \text{(S7)}$$

where I_ω and $I_{2\omega}$ are the intensities of fundamental and SHG light waves, respectively, *l* the thickness of the films (note that it is well below the coherent length in this study), *d* the effective second-order nonlinear coefficient, n_ω and $n_{2\omega}$ the refractive indices at the fundamental and SHG wavelengths, 800 nm and 400 nm, respectively. The refractive index of the superlattice film was estimated using the refractive index of $PbTiO_3$, ~2.9 at 400 nm and ~2.6 at 800 nm [2], comparing to $LiNbO_3$ (~2.5 at 400 nm and ~2.3 at 800 nm) [3].

To calculate the second-order nonlinear coefficient and the SHG efficiency of the $PbTiO_3/SrTiO_3$ superlattice, we measured the SHG intensities of the superlattice samples and a 300 nm thick *x*-cut LNOI ($LiNbO_3$ on insulator) film under the φ = 0° (*p*-in) and α = 0° (*p*-out) conditions using the same 45° incidence setup for direct comparison. The *x* axis of the LNOI film was aligned parallel with the plane of incidence, in which case the effective second-order nonlinear coefficient was taken as ~31 pm V^{-1} [4] calculated from χ_{33}. The maximum EFISH intensity was measured to be 1.39×10^5 cps for the superlattice under an input power of 150 mW, and 8.81×10^6 cps for LNOI under an input power of 50 mW. The measured SHG intensity for $LiNbO_3$ is shown in Figure S4a.

Given that the fundamental light was focused onto the $SrRuO_3$ top electrodes during the EFISH measurements, the transmittance of the $SrRuO_3$ electrode for the fundamental and second harmonic lights significantly affects the measured SHG intensity. To calibrate the influence of the $SrRuO_3$ electrode, a 30 nm thick $PbTiO_3$ film grown on a $SrRuO_3$ buffered LSAT substrate with 20 nm thick $SrRuO_3$ top electrode was prepared as a reference sample. Figure S4b compares the SHG intensity inside and outside the $SrRuO_3$ electrode areas under the same 45° incidence setup. According to the fitted parameters, the SHG intensity inside the $SrRuO_3$ electrode declines to ~1/8 of that outside under the same excitation power, independent of the input polarization states. Therefore, for the $SrRuO_3$/superlattice/$SrRuO_3$ capacitors, the measured SHG intensity is expected to be ~1/8 of the SHG intensity generated by the superlattice itself. This proportion factor is used to correct the measured SHG intensity for nonlinear coefficient calculations.

According to the above intensities, the maximum effective second-order nonlinear coefficient of the whole superlattices is determined to be 13.8 pm V^{-1}. Considering that the transitions of polar skyrmions only occur in $PbTiO_3$ layers, which are the effective polarization layers and only occupy about half the total thickness of the superlattices, the effective second-order nonlinear coefficient of the $PbTiO_3$ layers should be 29.5 pm V^{-1}. According to the calculations in Supplementary Note 1, under the $\varphi = 0°$ (p-in) and $\alpha = 0°$ (p-out) conditions, the effective second-order nonlinear coefficient χ of the superlattice films can be expressed as:

$$\chi = \frac{\sqrt{2}}{4}(\chi_{31} + \chi_{33} - 2\chi_{15}) \qquad \text{(S8)}$$

According to the proportionality of χ_{31}, χ_{33} and χ_{15} given in Supplementary Note 1, it follows that maximum χ_{33} = 54.2 pm V^{-1} in the $PbTiO_3$ layers, which is significantly higher than the χ_{33} of $PbTiO_3$ bulk single crystals (17 pm V^{-1} according to literature[2]).

The SHG efficiency can be expressed as

$$\eta = \frac{I_{2\omega}}{I_{\omega}^2} \qquad \text{(S9)}$$

where I_{ω} and $I_{2\omega}$ are the intensities of fundamental waves and SHG light waves, respectively. The SHG efficiency calculated from the measured intensity counts and sensitivity parameter of the PMT (Hamamatsu CH326, 4.4×10^5 s^{-1} pW^{-1} at 400 nm) is ~9.3×10^{-11} W^{-1} taking the reduction by the top $SrRuO_3$ layer into account. The SHG efficiency can also be estimated by comparing the SHG intensity with $LiNbO_3$; for the latter, the conversion efficiency was taken as ~1.45×10^{-8} W^{-1} for a crystal with 500 nm thickness [5] (then in our case, ~5.22×10^{-9} W^{-1} for the 300 nm thick LNOI films). The SHG efficiency of the superlattices is thus calculated to be ~6.07×10^{-11} W^{-1}, reasonably close to the efficiency calculated directly from the parameters of PMT. In conclusion, the SHG efficiency of the superlattices falls in between ~6.0-9.3×10^{-11} W^{-1}.

Supplementary Note 3 | The polarization autocorrelation analysis of the phase-field model.

The polarization distribution in the superlattice layer of the simulated phase-field model box was divided into a matrix of 192×192×256, with each grid corresponding to the size of a unit cell. The topological charge density, or Pontryagin density q is calculated from the polarization extracted from the model as:

$$q = \mathbf{P} \cdot \left(\frac{\partial \mathbf{P}}{\partial x} \times \frac{\partial \mathbf{P}}{\partial y}\right) \qquad \text{(S10)}$$

which reflects the topological nature of skyrmion systems. The autocorrelation function of the Pontryagin density q for both x and y directions were calculated as:

$$R_{qq}(u, v) = \frac{\Sigma(q(x,y,z)-E(q))(q(x+u,y+v,z)-E(q))}{n\,\sigma(q)} \qquad \text{(S11)}$$

$$R_x(q,t) = R(q,(t,0)) \quad \text{(S12)}$$

$$R_y(q,t) = R(q,(0,t)) \quad \text{(S13)}$$

$$R(q,t) = \sqrt{\frac{R_x(q,t)^2 + R_y(q,t)^2}{2}} \qquad \text{(S14)}$$

where $E(q)$ and $\sigma(q)$ are the average value and variance of q, n the total number of the cells, (u, v) the shift vector, and t the shift distance for autocorrelation calculation. A large $R(t)$ value indicates high similarity in the polarization distribution over the distance t. Therefore, the spatial frequency analysis of $R(t)$ can reveal the real-space modulation of the in-plane polarization. The autocorrelation functions along the x and y directions are shown in Fig. S6. A ~0.190 nm^{-1} correlation length is found in the superlattices with no external voltage applied, and a ~0.103 nm^{-1} correlation length is found at positive voltages. As the skyrmion walls on both sides are marked by non-zero Pontryagin density q, the 0.103 nm^{-1} and 0.190 nm^{-1} correlation lengths indicate the period of the skyrmions and the distance between the domain walls, respectively. A coexistence of the two correlation lengths means that the size of the skyrmions equates the distance between neighboring skyrmions. The centrosymmetry in the absence of external voltages originates from such equality in the distance, and the disappearance of the ~0.190 nm^{-1} correlation means a breaking of the centrosymmetry.

Supplementary Note 4 | The origin of SHG from the skyrmion walls.

The skyrmions in the $PbTiO_3/SrTiO_3$ superlattice consist of mainly Néel-type domain walls, whose SHG susceptibility is described in previous research[6] as:

$$\chi^{\text{Néel}} = \begin{pmatrix} \sin\delta & \cos\delta & 0 \\ -\cos\delta & \sin\delta & 0 \\ 0 & 0 & 1 \end{pmatrix} \begin{pmatrix} \chi_{11} & \chi_{12} & \chi_{13} & 0 & \chi_{15} & 0 \\ 0 & 0 & 0 & \chi_{24} & 0 & \chi_{26} \\ \chi_{31} & \chi_{32} & \chi_{33} & 0 & \chi_{35} & 0 \end{pmatrix}$$

$$\times \begin{pmatrix} \sin^2\delta & \cos^2\delta & 0 & 0 & 0 & -\sin 2\delta \\ \cos^2\delta & \sin^2\delta & 0 & 0 & 0 & \sin 2\delta \\ 0 & 0 & 1 & 0 & 0 & 0 \\ 0 & 0 & 0 & \sin\delta & \cos\delta & 0 \\ 0 & 0 & 0 & -\cos\delta & \sin\delta & 0 \\ \frac{1}{2}\sin 2\delta & -\frac{1}{2}\sin 2\delta & 0 & 0 & 0 & -\cos 2\delta \end{pmatrix} \quad \text{(S15)}$$

where δ is the orientation of domain walls. For the skyrmion with circular domain walls, the SHG susceptibility is the average of above susceptibilities $\chi^{\text{Néel}}$ within the range of δ from 0° to 360°, which is:

$$\chi_{\text{skyrmion}}^{\text{Néel}} = \frac{\int_0^{2\pi} \chi^{\text{Néel}} \mathrm{d}\delta}{2\pi} =$$

$$\begin{pmatrix} 0 & 0 & 0 & 0 & (\chi_{15}+\chi_{24})/2 & 0 \\ 0 & 0 & 0 & (\chi_{15}+\chi_{24})/2 & 0 & 0 \\ (\chi_{31}+\chi_{32})/2 & (\chi_{31}+\chi_{32})/2 & \chi_{33} & 0 & 0 & 0 \end{pmatrix}$$

(S16)

This form is similar to that of a *c*-domain with the 4*mm* point-group symmetry. Therefore, under positive voltages, the SHG susceptibility of the superlattice can be divided into two parts, one originating from the increasing *c*-domain, and the other from the circular domain walls. The SHG response originating from the skyrmion walls are forbidden under zero electric fields because of the presence of pseudo-centrosymmetry over a large length scale (as described in the main text and in Supplementary Note 3), and is insignificant at negative voltages because the skyrmion walls are far sparser (as shown in Fig. 3d-f).

Supplementary Note 5 | Device simulation

The simulation for the Fresnel lens design was performed employing frequency domain finite-element-method solver in COMSOL Multiphysics software. A side-view 2D model was constructed, including the designed 45° tilted Fresnel lens composed of superlattice film rings, which appeared as aligned rectangles in this view. A 28×105 μm^2 region was defined as the simulation area where all boundaries were set as perfect absorber for both 400 and 800 nm light. The 800 nm light incident from the left boundary at 1 V/m electric field strength, propagating normally to the right, which is equivalent with the case where the light incident from the top and reflected to the right direction. For the SHG process, light field at 400 nm was also calculated for the model. An additional nonlinear polarization term was considered with magnitude $P_{NL} \propto \chi^{(2)} E_\omega E_\omega^*$, where $\chi^{(2)}$ is the effective second-order nonlinear coefficient. An external d.c. electric field was assumed to be applied on the film, resulting in positive (negative) $\chi^{(2)}$ for even (odd)-level rings. The absolute values of these coefficients were directly taken from the EFISH experiment results shown in the main text Fig. 2.

Supplementary References

1. Zu, R. *et al.* Analytical and numerical modeling of optical second harmonic generation in anisotropic crystals using ♯SHAARP package. *npj Comput Mater* **8**, 246 (2022).
2. Singh, S., Remeika, J. P. & Potopowicz, J. R. Nonlinear Optical Properties of Ferroelectric Lead Titanate. *Appl. Phys. Lett.* **20**, 135–137 (1972).
3. Zelmon, D. E., Small, D. L. & Jundt, D. Infrared corrected Sellmeier coefficients for congruently grown lithium niobate and 5 mol. % magnesium oxide-doped lithium niobate. *J. Opt. Soc. Am. B* **14**, 3319 (1997).
4. Hao, Z. *et al.* Second-harmonic generation using d_{33} in periodically poled lithium niobate microdisk resonators. *Photon. Res.* **8**, 311 (2020).
5. Guo, Q. *et al.* Ultrathin quantum light source with van der Waals $NbOCl_2$ crystal. *Nature* **613**, 53–59 (2023).
6. Cherifi-Hertel, S. *et al.* Shedding light on non-Ising polar domain walls: Insight from second harmonic generation microscopy and polarimetry analysis. *Journal of Applied Physics* **129**, 081101 (2021).
