# Supplementary material for: Giant electric field-induced second harmonic generation in polar skyrmions
Source: Nat Commun. 2024 Feb 14;15:1374. doi: 10.1038/s41467-024-45755-5 (PMC10866987; doi:10.1038/s41467-024-45755-5)
Supplement: Supplementary file 3 — Description of Additional Supplementary Files [file 41467_2024_45755_MOESM3_ESM.pdf]

Description of Additional Supplementary Files:

Supplementary Movie 1: Polarization vector map in a xy slice for positive bias from 0 V to 15.6 V (left), and negative bias from 0 V to - 15.6 V (right).

Supplementary Movie 2: Polarization vector map in a xz slice for positive bias from 0 V to 15.6 V (left), and negative bias from 0 V to - 15.6 V (right).
