# Supplementary figures and images for: Giant electric field-induced second harmonic generation in polar skyrmions
Source: Nat Commun. 2024 Feb 14;15:1374. doi: 10.1038/s41467-024-45755-5 (PMC10866987; doi:10.1038/s41467-024-45755-5)

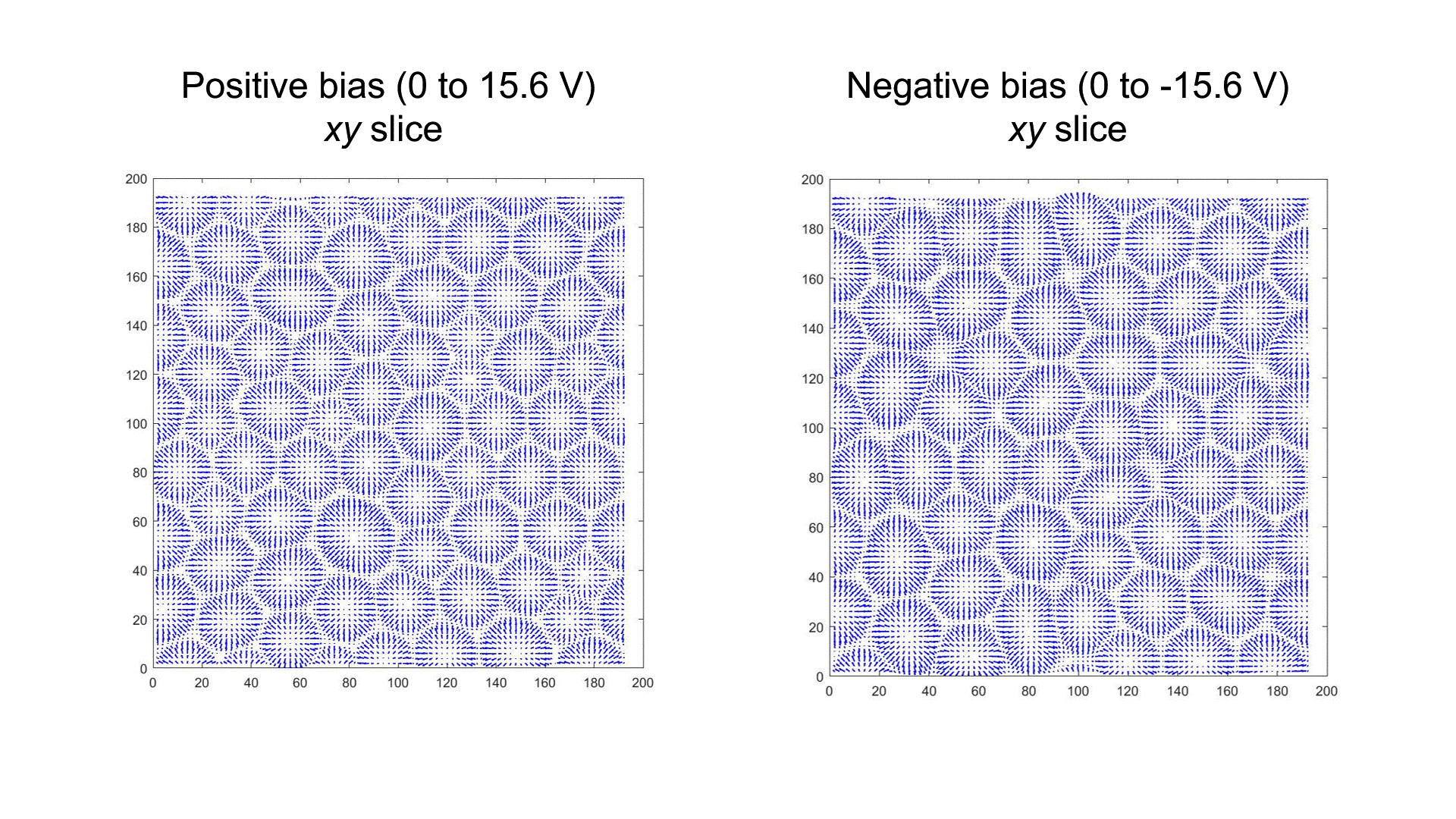

Supplement: Supplementary file 4 — Supplementary Movie 1 [file 41467_2024_45755_MOESM4_ESM.gif]

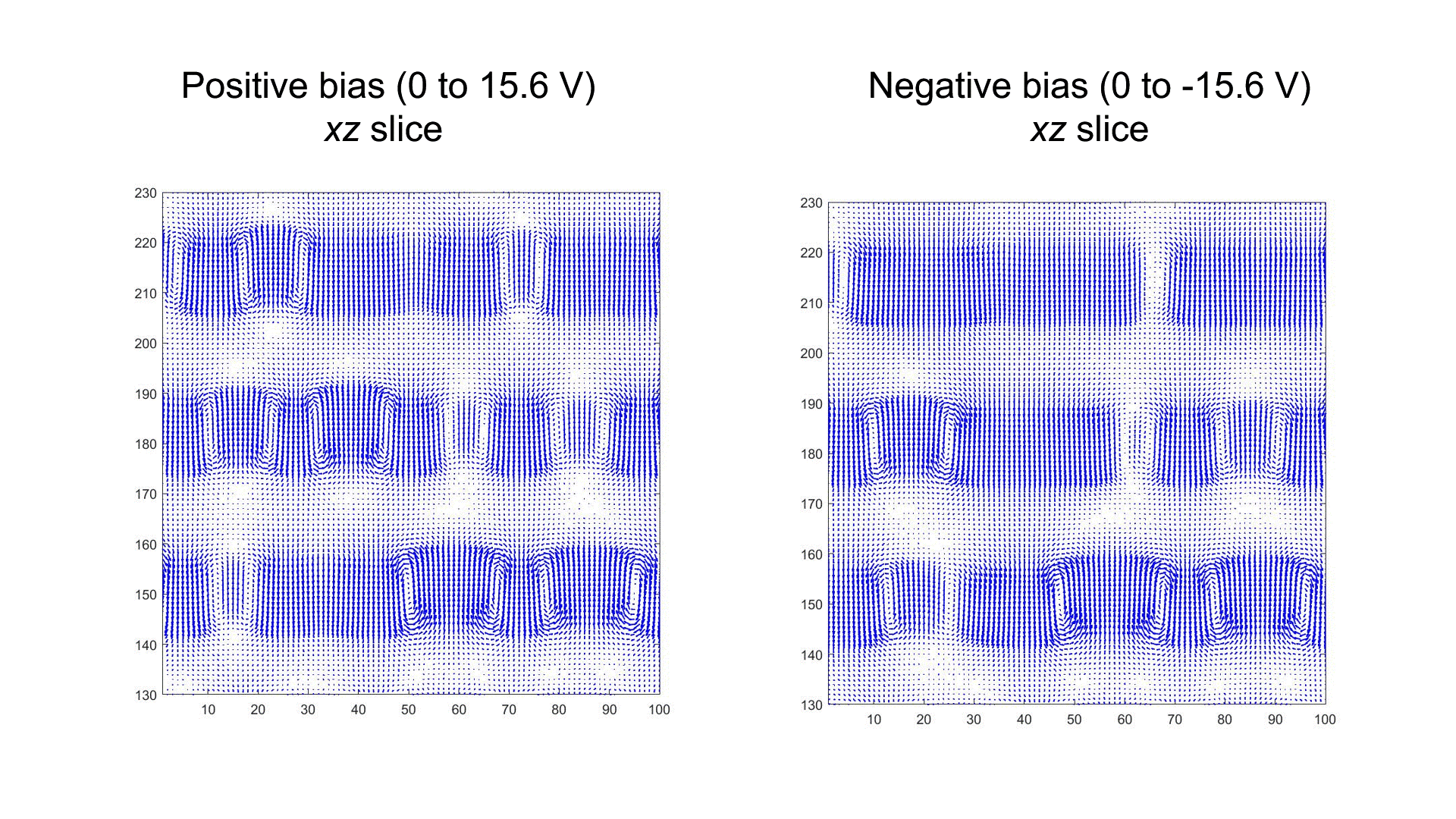

Supplement: Supplementary file 5 — Supplementary Movie 2 [file 41467_2024_45755_MOESM5_ESM.gif]
